# Upregulation of microRNA-224 is associated with aggressive progression and poor prognosis in human cervical cancer

**DOI:** 10.1186/1746-1596-8-69

**Published:** 2013-04-30

**Authors:** Shu-na Shen, Ling-feng Wang, Yong-feng Jia, Yu-qing Hao, Lin Zhang, Hui Wang

**Affiliations:** 1 Department of Gynaecology and Obstetrics, The Third Affiliated Hospital of Inner Mongolia Medical University, Baotou 014010, China; 2 Department of Burn, The Third Affiliated Hospital of Inner Mongolia Medical University, Baotou 014010, China; 3 Pathology Department, The First Affiliated Hospital of Inner Mongolia Medical University, Huhehot 010050, China; 4 Dermatological Department, The Third Affiliated Hospital of Inner Mongolia Medical University, Baotou 014010, China

**Keywords:** MicroRNA-224, Cervical cancer, Real-time quantitative RT-PCR assay, Prognosis

## Abstract

**Objective:**

Accumulating evidence for differential expression of microRNA-224 (miR-224) in various types of human cancer suggests that it may be play a crucial role in tumor biology. The previous microarray detection also shown that miR-224 was one of miRNAs with significant upregulation in cervical cancer tissues relative to adjacent normal tissues. However, little is known about the function of miR-224 in human cervical cancer. The aim of this study was to investigate the clinical significance of miR-224 expression in cervical cancer.

**Methods:**

MiR-224 expression in 126 pairs of fresh human cervical cancer and adjacent normal tissues was measured by real-time quantitative RT-PCR assay.

**Results:**

miR-224 expression was significantly upregulated in cervical cancer tissues when compared with corresponding adjacent normal tissues (P < 0.001). It was also significantly higher in the cancerous tissues of patients with advanced FIGO stage cervical cancer than those with early FIGO stage (P = 0.02). In addition, miR-224 was expressed at significantly higher levels in lymph node metastasis-positive patients than in lymph node metastasis-negative patients (P = 0.008). Moreover, we found that lesser differentiated tumors expressed higher miR-224 (P = 0.03). Finally, there were sufficient evidence to confirm its value in the status of vascular invasion (P = 0.01) and human papillomavirus (HPV) infection (P = 0.02) in cervical cancer. More importantly, Kaplan-Meier analysis showed that cervical cancer patients with high miR-224 expression tend to have shorter overall survival. In multivariate analysis stratified for known prognostic variables, miR-224 was identified as an independent prognostic marker.

**Conclusion:**

Our data indicated that miR-224 upregulation was associated with aggressive progression and poor prognosis in cervical cancer. MiR-224 was identified for the first time as an independent marker for predicting the clinical outcome of cervical cancer patients.

**Virtual slides:**

The virtual slide(s) for this article can be found here: http://www.diagnosticpathology.diagnomx.eu/vs/2170449349527493

## Introduction

Cervical cancer is the third most common malignancy among women worldwide, with an estimated global incidence of over 500,000 new cases and tremendously high death cases of 260,000 annually [1]. It is the result of a multistep process that involves the transformation of normal cervical epithelium to preneoplastic cervical intraepithelial neoplasia that is subsequently transformed to invasive cervical cancer [2]. Early detection and diagnosis have become accurate and inexpensive through routine papanicolaou tests, and the recent advent of human papillomavirus (HPV) vaccine may have a significant impact on prevention of cervical cancer which is strongly associated with infection and subsequent transformation of cervical cells by specific HPV subtypes [3]. Although radiotherapy, chemotherapy and surgery have been recently used as standard treatment modalities for patients with cervical cancer, with consequent disease remission, clinical outcomes vary significantly between patients and can be difficult to predict. Therefore, it is important to understand the complete knowledge of the molecular biology, genetics, causes and cellular origin of cervical cancers which are of value in the development of improved therapeutic strategies and in the identification of prognostic markers.

MicroRNAs (miRNAs) are short non-coding RNAs that were initially discovered in the early 1990s and generally control gene expression at the posttranscriptional level through mRNA degradation and/or translational repression [4]. miRNAs bind to the 3’ untranslated regions (3’-UTR) of their target mRNAs, mediating translational repression and/or mRNA degradation [5]. Thousands of miRNAs have been identified in nematodes, insects, birds, amphibians, fishes, plants, mammals, and even viruses using molecular cloning and bioinformatics prediction strategies [6]. Recent studies have suggested and reinforced their roles as important regulators of gene expression in a broad range of physiological and pathological processes, including cancer development and progression. Aberrant expression levels of miRNAs have been demonstrated to be involved in several forms of solid tumors such as hepatocellular carcinoma, colorectal cancer, prostate cancer, cervical cancer, breast cancer, ovarian carcinoma, and lung cancer [7-10]. They function either as tumor suppressors or oncogenes according to the roles of their target genes. Changes in their expression are able to be used as robust and important biomarkers for cancer risk, diagnosis and prognosis, even as miRNA-based therapeutic targets with a great interest. For example, several of miRNAs, such as miR-143, miR-145, miR-21, miR-424 and miR-205 have consistently been reported as dysregulated in cervical cancer [11-15]. Especially, Wang et al. [11] found that miR-143 and miR-145 were underexpressed in both cervical cancer tissues and HPV-infected tissues with pre-neoplastic lesions, suggesting that their reduction take place in an early step before cancer development. Xu et al. [13] identified a crucial tumor suppressive role of miR-424 in the progression of cervical cancer at least partly via upregulating the expression of Chk1 and p-Chk1, suggesting that miR-424 may be a candidate of prognostic predictor or an anticancer therapeutic target for cervical cancer patients.

In the previous study of Rao et al. [16], using MicroRNA array, they detected differential expression of miRNAs in human cervical cancer tissues relative to adjacent normal cervical tissues. In cervical cancer tissues, miR-224 was found to be one of the upregulated miRNAs (2.7221-fold higher than in adjacent normal cervical tissues). However, little is known about the function of miR-224 in human cervical cancer. The aim of this study was to investigate the clinical significance of miR-224 expression in cervical cancer.

## Materials and methods

### Patients and tissue samples

This study was approved by the Research Ethics Committee of the Third Affiliated Hospital of Inner Mongolia Medical University and the First Affiliated Hospital of Inner Mongolia Medical University, P. R. China. Written informed consent was obtained from all of the patients. All specimens were handled and made anonymous according to the ethical and legal standards.

Fresh cervical cancer and matched adjacent normal tissue specimens were collected from 126 patients who underwent surgery between May 2003 and August 2007 in the Third Affiliated Hospital of Inner Mongolia Medical University and the First Affiliated Hospital of Inner Mongolia Medical University. The corresponding adjacent normal tissues were obtained 3 cm beyond the boundary of cervical cancer tissues. The fresh tissue specimens were immediately frozen in liquid nitrogen until use. No patients had preoperative chemotherapy, radiotherapy, or other treatment history or other inflammatory diseases. The cervical cancer patients were aged from 26 to 68 years with a median of 50 years. Pathological diagnosis of all 126 cervical cancer patients was cervical squamous cell carcinoma. The degree of differentiation was well differentiated in 45 cases, moderately differentiated in 46 cases, and poorly differentiated in 35 cases. A total of 82 cases had lymph node metastases, while 44 cases did not have lymph node metastases. The clinical stage according to the International League of Gynecology and Obstetrics (FIGO, 2009) was 28 cases of stage Ib, 36 cases of stage IIa, 32 cases of stage IIb, and 30 cases of stage IIIa. A total of 64 stage Ib~IIa cases were grouped as early stage, and a total of 62 stage IIb~IIIa cases were grouped as late stage. The clinicopathologic features of all the patients were summarized in Table 1.

**Table 1:** Association between miR-224 expression and different clinicopathological features of human cervical cancers

**Clinicopathological features**	**No. of cases**	**miR-224 expression**		**P**
		**High (n, %)**	**Low (n, %)**	
**Age**				
<50	60	30 (50.0)	30 (50.0)	NS
≥50	66	36 (54.5)	30 (45.5)	
**Tumor size (cm)**				
<4.0	58	30 (51.7)	28 (48.3)	NS
≥4.0	68	36 (52.9)	32 (47.1)	
**Histological grades**				
Well differentiated	28	4 (14.3)	24 (85.7)	0.03
Moderately differentiated	32	12 (37.5)	20 (62.5)	
Poorly differentiated	66	50 (75.8)	16 (24.2)	
**FIGO stage**				
Ib~IIa	64	20 (31.3)	44 (68.7)	0.02
IIb~IIIa	62	46 (74.2)	16 (25.8)	
**Lymph node metastasis**				
No	79	24 (30.4)	55 (69.6)	0.008
Yes	47	42 (89.4)	5 (10.6)	
**Vascular invasion**				
No	72	20 (27.8)	52 (72.2)	0.01
Yes	54	46 (85.2)	8 (14.8)	
**HPV**				
(−)	48	10 (20.8)	38 (79.2)	0.02
(+)	78	56 (71.8)	22 (28.2)	

Clinical follow-up was available for all patients (median, 51.9 months). Overall survival time was calculated from the date of the initial surgical operation to death. Patients, who died of diseases not directly related to their cervical cancers or due to unexpected events, were excluded from this study. Follow-up information of all patients was updated every 3 months for the first 2 years, every 4 months for the third year, every 6 months for the fourth and fifth years, and then every year thereafter by telephone visit and questionnaire letters. Death of the participants was ascertained by reporting from the family and verified by review of public records.

### Real-time quantitative RT-PCR for miRNA

Real-time quantitative RT-PCR for miRNA was performed to detect the expression levels of miR-224 in human cervical cancer and matched adjacent normal tissues. Total RNA was extracted from tissue samples of 126 pairs of cervical cancer and adjacent normal tissues using TRIzol (Invitrogen) according to the manufacturer’s protocol. RNU6B was used as internal control. The specific cDNA of miR-224 and RNU6B were synthesized from total RNA using gene-specific primers according to the TaqMan MicroRNA assays protocol (Applied Biosystems, Foster City, CA, USA). Reverse transcriptase reactions contained 10 ng of total RNAs, 50 nmol/l stem-loop RT primer, 1× RT buffer, 0.25 mmol/l each of deoxynucleotide triphosphate (dNTP), 3.33 U/μl MultiScribe reverse transcriptase, and 0.25 U/μl RNase Inhibitor. The 20-μl reaction volumes were incubated in Bio-Rad i-Cycler (Bio- Rad Laboratories, Hercules, CA, USA) in a 96-well plate for 30 min at 15°C, 30 min at 40°C, 5 min at 85°C, and then held at 4°C. Real-time PCR was performed using an Applied Biosystems 7500 real-time PCR system. The reaction mixture (10 μL total volume per well) included 2 ng cDNA, 1× TaqMan Universal PCR master mix, and 1 μl of primers and probe mix of the TaqMan MicroRNA Assays. Relative quantification of target miRNA expression was evaluated using the comparative cycle threshold (CT) method. The raw data were presented as the relative quantity of target miRNA, normalized with respect to RNU6B. Each sample was examined in triplicate. Mean normalized gene expression ± standard deviation (SD) was calculated from three independent experiments.

### Statistical analysis

All computations were carried out using the software of SPSS version13.0 for Windows (SPSS Inc, IL, USA). Data were expressed as mean ± SD. The analysis of variance (ANOVA) was used to determine the statistical differences among the groups. The Kaplan-Meier method was used to estimate survival rates, and the log-rank and the Wilcoxon rank sum tests were used to assess survival differences between groups. The Cox proportional hazards model for multivariate survival analysis was used to assess predictors related to survival. Differences were considered statistically significant when *p* was less than 0.05.

## Results

### MiR-224 upregulation in human cervical cancer

**Figure 1:**
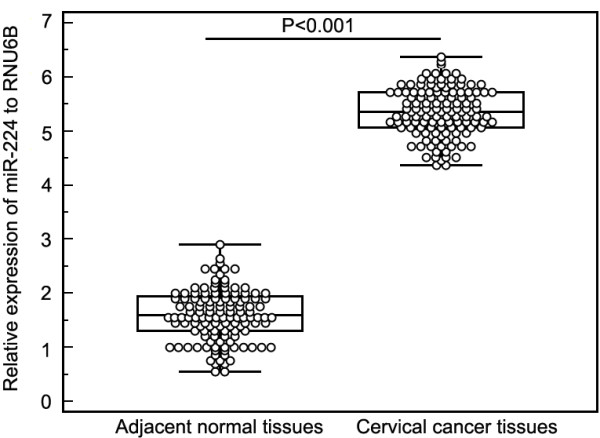
**MiR-224 expression in 126 pairs of cervical cancer and adjacent normal tissues were respectively detected by real-time quantitative RT-PCR assay.** After normalization to RNU6B, the expression level of miR-224 in cervical cancer tissues (mean ± SD: 9.8 ± 0.9) was significantly higher than that in adjacent normal tissues [mean ± SD: 3.2 ± 0.9, ratio (cervical cancer/normal controls) =3.1, P < 0.001].

MiR-224 expression was detected in 126 pairs of human cervical cancer and adjacent normal tissues by real-time quantitative RT-PCR. As shown in Figure 1, after normalization to RNU6B expression levels, the expression level of miR-224 in cervical cancer tissues (mean ± SD: 5.4 ± 0.9) was significantly higher than that in adjacent normal tissues (mean ± SD: 3.2 ± 0.9, P < 0.001). The median expression level of miR-224 (5.4) was used as a cutoff point to divide all 126 patients into two groups: cervical cancer patients who express miR-224 at levels less than the cutoff value were assigned to the low expression group (mean expression value 4.6, n = 60), and those with expression above the cutoff value were assigned to the high expression group (mean expression value 6.1, n = 66).

### MiR-224 upregulation associates with aggressive clinicopatholigcal parameters of human cervical cancer

Table 1 summarized the association between miR-224 expression and clinicopathological parameters in cervical cancers. miR-224 expression was significantly higher in the cancerous tissues of patients with advanced FIGO stage cervical cancer than those with early FIGO stage (P = 0.02, Table 1). In addition, miR-224 was expressed at significantly higher levels in lymph node metastasis-positive patients than in lymph node metastasis-negative patients (P = 0.008, Table 1). Moreover, we found that lesser differentiated tumors expressed higher miR-224 (P = 0.03, Table 1). Finally, there were sufficient evidence to confirm its value in the status of vascular invasion (P = 0.01, Table 1) and human papillomavirus (HPV) infection (P = 0.02, Table 1) in cervical cancer.

### MiR-224 upregulation associates with poor prognosis in patients with human cervical cancer

**Figure 2:**
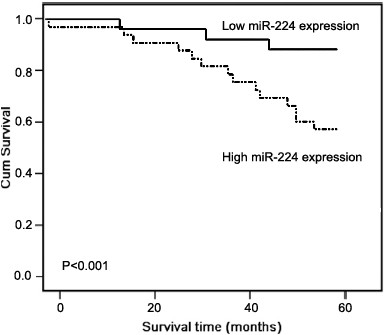
Kaplan-Meier curves for survival time in patients with cervical cancers divided according to miR-224 expression: significantly shorter survival times for patients with high miR-224 expression than for those with low miR-224 expression (P < 0.001).

The association between miR-224 expression and survival of cervical cancer patients was investigated by Kaplan–Meier analysis and log-rank test. During the follow-up period, 66 of the 126 patients (52.4%) had died. As shown in Figure 2, cervical cancer patients with high miR-224 expression tend to have shorter overall survival than those with low miR-224 expression (log-rank test: P < 0.001). Univariate analysis demonstrated that FIGO stage (P < 0.001), the status of lymph node metastasis (P = 0.006) and vascular invasion (P = 0.01), and miR-224 expression (P < 0.001) were significantly associated with overall survival of cervical cancer patients (Table 2). No significant associations were found for age at diagnosis, tumor size, histological grade, and HPV status and patient outcome. Multivariate analysis using the Cox proportional hazards model for all variables that were significant in the univariate analysis showed that FIGO stage (P = 0.01), the status of lymph node metastasis (P = 0.02) and vascular invasion (P = 0.04), and miR-224 expression (P = 0.009) were independent prognostic factors for patients with cervical cancer (Table 2).

**Table 2:** Univariate and multivariate analysis of prognostic parameters in patients with cervical cancers by Cox regression analysis

**Variables**	**Univariate log-rank test (P)**	**Cox multivariable analysis (P)**	**Relative risk (RR)**
**Age at diagnosis (years)**			
<50 vs. ≥50	0.3	0.7	0.6
**Tumor size (cm)**			
<4.0 vs. ≥4.0	0.6	0.8	0.6
**FIGO stage**			
Ib~IIa vs. IIb~IIIa	<0.001	0.01	6.2
**Histological grades**			
Well ~ moderately differentiated vs. poor differentiated	0.08	0.10	1.3
**Lymph node metastasis**			
No vs. Yes	0.006	0.02	3.9
**Vascular invasion**			
No vs. Yes	0.01	0.04	2.7
**HPV**			
(−) vs (+)	0.06	0.1	1.1
**MiR-224 expression**			
High vs Low	<0.001	0.009	6.8

## Discussion

Cervical cancer remains one of the leading causes of cancer death in women worldwide [17]. In spite of the development of advanced therapeutic strategies, the prognosis in patients with this cancer varies significantly and is hard to predict. Treatment outcome still depends primarily on early detection and diagnosis. Recent studies have demonstrated that some abnormal molecular biology changes may play central roles in the tumorigenesis and the development of cervical cancer [18-24]. For example, Liu et al. [25] reported that genomic amplification of hTERC gene may be associated with more progressive cervical cancer; Imura et al. [26] demonstrated that Lam-5 was a useful biomarker in the evaluation of invasiveness in cervical adenocarcinoma. Therefore, it is critical to identify biomarkers for the early diagnosis and the early identification of patients with a high risk of treatment failures, in order to modify therapeutic methods for improving overall survival of patients with cervical cancer.

miR-224 is a family of miRNA precursors found in mammals, including humans. It was identified and ends mapped by cloning from Weri cells in human. The sequence of miR-224 maps to chromosome X [27]. Accumulating evidences for differential expression of miR-224 in various types of human cancer suggest that it may be play a crucial role in tumor biology. However, it is also characterized by contradictory properties, since it can promote or inhibit cancer cell growth, depending on the malignancy type. For example, miR-224 is one of the most commonly up-regulated miRNAs in hepatocellular carcinoma [28]. Overexpression of miR-224 in liver cells was reported to increase cell proliferation and apoptosis as well as cell migration and invasion [29]. Up-regulated miR-224 was also identified in a subtype of medulloblastomas. Exogenous expression of miR-224 was found to inhibit proliferation, increase radiation sensitivity and reduce anchorage-independent growth of medulloblastoma cells [30]. In breast cancer cell lines, Huang et al. [31] found that the overexpression of miR-224 which plays an important role in metastasis of cancer cells to the bone by directly suppressing the RKIP tumor suppressor. In addition to these, the upregulation of miR-224 was also shown in aggressive pancreatic ductal adenocarcinoma [32], clear cell renal cell carcinoma [33], thyroid cancer [34] and bladder cancer [35]. In contrast, a downregulation of miR-224 has been observed in ovarian cancer [36], prostate cancer [37] and oral carcinoma [38].

Observations mentioned above of upregulation or downregulation of miR-224 in different tumor types suggest that miR-224 may have different functions in cancer development depending on the cell type involved. Previous study has shown miR-224 to have increased expression in cervical cancer [16]. For a more comprehensive insight into the clinical value of miR-224, we in the present study performed a real-time quantitative RT-PCR assay to explore the expression profile of this miRNA as well as to investigate its association with clinicopathological features of cervical cancer patients. Our data proved that miR-224 expression was significantly higher in cervical cancer compared with that in adjacent normal tissues. In addition, miR-224 expression was also proved to be associated with histological grade, FIGO stage, HPV status, lymph node metastasis, and vascular invasion for high miR-224 expression was more frequently detected in cervical cancer with poor differentiation, advanced FIGO stage, positive HPV infection, lymph node metastasis, and vascular invasion, which strongly suggested that miR-224 was involved in the invasion and metastasis of cervical cancer. More importantly, we proved that miR-224 expression was significantly associated with overall survival of patients with cervical cancer. In support of this, Kaplan-Meier analysis of overall survival showed that patients with tumors of high miR-224 expression tend to have a significantly shorter overall survival compared with patients whose tumors do not, indicating that high miR-224 expression is a marker of poor prognosis for patients with cervical cancer. Cox proportional hazards model adjusted for known prognostic variables such as histological grade, FIGO stage, HPV status, lymph node metastasis, and vascular invasion proved that miR-224 was an independent prognostic marker for cervical cancer. Thus, miR-224 could be used as a molecular prognostic marker additive to the known prognostic indicator, in order to identify patients who are more likely to have higher risk of death, thus, should receive more aggressive treatment.

In conclusion, our data indicated that miR-224 upregulation was associated with aggressive progression and poor prognosis in cervical cancer. MiR-224 was identified for the first time as an independent marker for predicting the clinical outcome of cervical cancer patients.

## Competing interests

The authors declare that they have no competing interests.

## Authors’ contributions

SS carried out the real-time quantitative RT-PCR assay and drafted the manuscript; WL, JY, and HY participated in the real-time quantitative RT-PCR assay and data analysis; ZL drafted the manuscript; WH designed the study and revised the manuscript. All authors read and approved the final manuscript.
